# Hepatocyte Turnover in Chronic HCV-Induced Liver Injury and Cirrhosis

**DOI:** 10.1155/2015/654105

**Published:** 2015-03-29

**Authors:** Nikolaos P. Karidis, Ioanna Delladetsima, Stamatios Theocharis

**Affiliations:** First Department of Pathology, Medical School, National and Kapodistrian University of Athens, 75 Mikras Asias Street, 11527 Athens, Greece

## Abstract

Chronic hepatitis C virus (HCV) infection may eventually lead to progressive liver fibrosis and cirrhosis through a complex, multistep process involving hepatocyte death and regeneration. Despite common pathogenetic pathways present in all forms of liver cirrhosis irrespective of etiology, hepatocyte turnover and related molecular events in HCV-induced cirrhosis are increasingly being distinguished from even “similar” causes, such as hepatitis B virus- (HBV-) related cirrhosis. New insights in HCV-induced hepatocellular injury, differential gene expression, and regenerative pathways have recently revealed a different pattern of progression to irreversible parenchymal liver damage. A shift to the significant role of the host immune response rather than the direct effect of HCV on hepatocytes and the imbalance between antiapoptotic and proapoptotic signals have been investigated in several studies but need to be further elucidated. The present review aims to comprehensively summarize the current evidence on HCV-induced hepatocellular turnover with a view to outline the significant trends of ongoing research.

## 1. Introduction

Liver cirrhosis is the end-stage condition of a multistep process, initiating from a primary hepatotoxic effect and resulting in insufficiency of liver function due to overwhelming hepatocyte damage. Although the functional reserve of the liver is the highest among all other organs, the cirrhotic liver rapidly loses its ability to compensate the chronic effect of a causative agent with significant biochemical and physiological changes for the organism. Apart from being a highly morbid and eventually lethal condition, cirrhosis also provides the most suitable substrate for hepatocellular cancer (HCC) development [1]. In fact, most HCC cases occur in cirrhotic liver, regardless of etiology, implying the effect of common molecular and cellular pathways that lead to carcinogenesis.

Dysregulation of hepatocellular death and regeneration, that is, hepatocyte turnover, in chronic liver damage seems to play a major role in the pathophysiology of liver cirrhosis and recent research has focused on the events responsible for the initiation and progress of irreversible alterations in liver tissue [2]. However, liver regeneration in cirrhosis does not seem to follow the same pattern as in normal liver tissue. An imbalance in the damage-regeneration sequence and distinct molecular pathways are considered important for the development of cirrhosis and subsequent oncogenesis [3].

Despite common pathophysiological aspects of liver cirrhosis, significant variations in gene expression and hepatocyte turnover have been revealed for the different etiologic factors. Differences have become evident even between previously thought “similar” causes, such as hepatitis B virus (HBV) and hepatitis C virus (HCV) [4]. Studies on the pathobiology of these viruses and the pathophysiology of chronic hepatitis with regard to the primary cause have suggested that chronic liver injury may lead to fibrosis and cirrhosis through distinct molecular and cellular events. Several novel genes whose dysregulation might play a role in the earliest stages of HCC development have been discovered in the last two decades [5]. The pathophysiology of acute and chronic liver damage induced by HBV has been so far more thoroughly investigated and a cell-mediated immune response to HBV gene products on the hepatocellular membrane is considered the major cause of liver injury. The mechanisms of liver damage and oncogenesis related to HCV are less understood and current research has focused on the differential gene expression in chronic HCV infection [6].

The aim of the present review is to extract and present current evidence on chronic hepatitis mediated by HCV (CHC), with regard to the unique alterations of hepatocyte turnover and the distinct molecular events, which lead to liver fibrosis and cirrhosis.

## 2. Basic Concepts of Liver Regeneration in Cirrhosis

Liver regeneration has been studied in several experimental protocols with the two-third partial hepatectomy in the rat being the most commonly used. Regeneration is an early term since the excised liver lobes do not actually grow back and residual liver parenchyma exhibits a compensatory growth. The process of liver regeneration involves at least three major pathways: (i) the cytokine pathway, a priming process that forces hepatocytes into the cell cycle, (ii) the growth factor pathway, which promotes cell cycle progression, and (iii) the metabolic pathway, which leads to hepatocyte growth and proliferation. Complex interactions between the active components of these pathways exist in a manner where lack of any regenerative factor may delay but not completely suppress regeneration. The role of each component of regeneration may be significantly altered in cirrhotic liver compared to normal tissue. The concept of “irregular regeneration,” that is, repeated cycles of regeneration after ongoing hepatocellular damage which induce multiple populations of hepatocytes differing in age, growth potential, and functional activity within a regenerative nodule, has been long associated with chronic viral hepatitis (CVH) and cirrhosis [7]. In HCV-mediated chronic liver damage and cirrhosis, particularly, irregular regeneration has recently been the target of interferon (IFN) therapy. Responders to IFN have shown improvement in the pattern of irregular regeneration while nonresponders exhibited an increased risk for HCC development [8].

Regeneration in cirrhotic liver after partial hepatectomy demonstrates an initial acceleration, although the extent of regeneration is inadequate due to early cessation of the proliferative process. The peak expression of the transforming growth factor- (TGF-) beta 1, potent regulator of liver regeneration, coincides with the peak expression of the hepatocyte growth factor (HGF), potent mitogen of hepatocytes* in vitro* and* in vivo*, instead of anteceding it [9]. A decrease in cytokine expression promoting liver growth, such as interleukin- (IL-) 6 and tumor necrosis factor- (TNF-) alpha, has also been implicated in impaired regeneration of cirrhotic liver. Interestingly, the expression of HGF was not significantly different from normal, although the expression of the c-Met/HGF receptor was strongly suppressed [10]. Despite intact expression of growth factors responsible for regeneration, the regenerative capacity of the cirrhotic liver is overall reduced. Studies on the role of the transcriptional activators CCAT/enhancer-binding protein (C/EBP) and activating protein- (AP-) 1, which modulate cell cycle regulators, showed that impaired activities of these factors in cirrhosis lead to suppression of regeneration after partial hepatectomy through downregulation of cyclin-D1, cyclin-E, and cyclin-A expression [11]. The telomere hypothesis for liver fibrosis and cirrhosis has gained significant popularity in the last decade. Telomerase-deficient mice lacking the RNA component of telomerase (mTERC −/−) showed significantly impaired regenerative capacity after partial hepatectomy, acute or chronic liver injury [12, 13]. Hepatocytes with critically short telomeres are not allowed to reenter the cell cycle. Therefore, cells with adequate proliferative activity, that is, sufficient telomere length, are forced to undergo additional rounds of cell division, which gradually lead to further telomere shortening, thus accelerating the imbalance between proliferative and nonproliferative cells [14]. Angiogenic factors have also been implicated in liver regeneration in both normal and cirrhotic livers. In particular, vascular endothelial growth factor (VEGF) and its receptors (VEGFRs) are involved in both liver fibrosis and cirrhotic remodeling [15]. Hepatocellular hypoxia due to impaired sinusoidal permeability in cirrhosis [16] is the primary stimulus for VEGF expression. During liver regeneration, a complex spatiotemporal pattern of VEGFRs' expression in endothelial cells has been observed, suggesting a significant, yet not clearly defined, role for VEGF in cirrhosis [17]. Endostatin, an endogenous inhibitor of angiogenesis and tumor growth, has been investigated for its role in regeneration in cirrhotic liver. Serum endostatin levels in hepatectomized cirrhotic liver showed a significant increase compared to normal liver tissue which, however, was not associated with regenerative capacity as in normal liver [18]. This finding strongly suggests a dysregulation of normal regenerative pathways in liver cirrhosis.

The crucial role of regenerative alternatives in cases of severe liver injury when hepatocytes lose their proliferative capacity has also been underlined in several studies. Hepatic “oval” cells with mixed biliary and hepatocytic gene expression patterns proliferate intensely in the periportal areas of the hepatic lobule in an effort to maintain adequate functional liver tissue [19]. Through transformation to basophilic and eventually mature hepatocytes, they interact with hepatic stellate cells (HSCs) which finally produce HGF, fibroblast growth factor- (FGF-) 1, FGF-2, and VEGF [20]. Interestingly, “oval” cells normally do not exist in the liver and they emerge as a “progenitor” cell population under circumstances of extreme liver injury [21]. HSCs, which comprise a mesenchymal liver cell population, also respond to chronic hepatocellular injury by expressing proteins known as morphogens. Among these, Wnt and Hedgehog (Hh) signaling seem to be directly implicated in liver regeneration by interacting with the hepatocellular reserve through *β*-catenin and HGF expression [22, 23]. However, the role of HSCs in chronic liver injury still remains unclear as they may also contribute to fibrogenesis in response to hepatocellular damage [24].

## 3. Molecular Events of Hepatocyte Turnover Specific to HCV-Mediated Liver Cirrhosis

Chronic liver damage mediated by the two most common viral agents, that is, HBV and HCV, shares several common pathological features, although their virologic profile and pathobiology are completely different. In general, progression of viral hepatitis may lead to chronic hepatitis, cirrhosis, and, in some instances, HCC. Despite the common end stages of liver damage, epidemiologic studies have suggested that the mode of disease progression differs between HBV and HCV infections [4, 5]. The underlying mechanisms have only recently been investigated in an effort to reveal the distinct characteristics of hepatocellular turnover and regeneration with possible therapeutic implications. Studies so far have mainly focused on HBV-mediated cirrhosis and little is yet known about the specific features of HCV infection.

### 3.1. HCV-Induced Hepatocellular Injury

HCV-mediated hepatocellular injury has been extensively investigated in order to elucidate the primary effect of HCV on the hepatocyte, which is thought to influence the sequence of cellular and molecular events leading to irreversible parenchymal damage. Moreover, hepatocellular damage seems to be a major stimulus for regeneration in both normal and cirrhotic liver. Whether HCV is directly cytopathic or its detrimental effect is immunologically mediated has been a matter of debate among researchers. A direct cytopathic effect was suggested in early studies [25]; however, a persistent intrahepatic Th1 immune response and the role of numerous Th1-associated cytokines in CHC have been more recently documented [26, 27]. A selective increase in chemokines and chemokine receptors that promote the accumulation of Th1 cells, such as CXC chemokine receptor- (CXCR-) 3 and its ligands CXCL10 and CXCL9, has also been observed in CHC [27]. Despite the prominent activation of Th1 cells, their response is inadequate to eradicate the virus and represents a nonspecific chronic inflammatory response which eventually leads to chronic liver injury [28, 29]. Apart from maintaining chronic inflammation, T cells also play a significant role in promoting hepatocellular apoptosis in HCV-related cirrhosis. Significantly increased proportions of memory T cells (CD3^+^ CD45RO^+^), Fas^+^ T cells (CD3^+^ CD95^+^, CD4^+^ CD95^+^, and CD8^+^ CD95^+^), and NK T cells in portal venous blood were found in adult cirrhotic patients [30]. An enhanced Fas/FasL-mediated apoptotic death of hepatocytes as well as an imbalance between hepatocellular apoptosis and regeneration is attributed to the activation of these immune cells. Moreover, the portal venous levels of IL-6 showed a significant increase in HCV-related cirrhosis [30]. The complex of IL-6 and its soluble receptor IL-6R has been directly implicated in the liver regeneration process and a possible antiapoptotic effect has been speculated, although not yet fully clarified [31].

### 3.2. Differential Gene Expression in Chronic HCV-Mediated Liver Injury and Cirrhosis

Two landmark studies have focused on differential gene expression in the pathogenesis of chronic hepatitis B- (CHB-) and CHC-induced liver injury, in an attempt to discriminate the molecular events which lead to cirrhosis and HCC development [32, 33]. The complementary DNA (cDNA) microarray gene profiling technique was implemented in CHC samples in the former study and HCV-induced cirrhosis in the latter. In the first study by Honda et al. [32], several genes, such as those coding for interferon-*α*, interferon regulatory factor 7, cytoplasmic dynein light chain, and acute myeloid leukemia 1 protein, were upregulated in both CHB and CHC lesions. Interestingly, the gene expression pattern of CHC-related lesions differed significantly from that of CHB and was further associated with the progress of disease. In mild to moderate CHC, Dr-nm23 (nonmetastatic cells protein 3 or NME3) and type II plasminogen activator inhibitor genes were upregulated, while cell division cycle 42 GTP-binding protein (CDC42) gene was downregulated. Dr-nm23 inhibits granulocyte differentiation and promotes apoptosis [34], while deficiency of CDC42 has been shown to induce chronic liver injury and promote hepatocarcinogenesis [35]. Furthermore, upregulation of Bcl-2 and Bcl-w genes, which inhibit apoptosis and promote cell proliferation, was characteristic of CHC. With regard to genes involved in the cell cycle, thus in liver regeneration, E2F transcriptional factor and members of the MAP kinase family as well as NF-*κ*B were upregulated in CHC, suggesting acceleration of hepatocellular proliferation via an antiapoptotic effect. Conversely, proapoptotic and cell-cycle suppressor genes were upregulated in CHB. Finally, tissue metalloproteinase inhibitor gene, which controls cell-cell interaction and maintenance of extracellular matrix (ECM), was significantly upregulated in CHC lesions [32], suggesting predominance of an anti-inflammatory response to HCV chronicity. In the second study by Shackel et al. [33], intrahepatic differential gene expression was investigated in a similar way in CHC cirrhosis compared to autoimmune hepatitis-associated cirrhosis and normal liver tissue. A remarkable difference was shown in the expression of apoptosis-related genes in CHC cirrhosis as well as in the persistence of Th1-associated inflammatory response. The expression of connective tissue growth factor (CTGF), cyclin-dependent kinase inhibitor 1C (CDKN1C), cytokine receptor EB13, transducer of ERBB-2 (TOB), and secreted apoptosis-related protein 3 (SARP3; sFRP5) genes was specifically upregulated in CHC cirrhosis, along with other apoptotic genes, such as Fas and TNF-*α*. Of these genes, the expression of SARP3, a proapoptotic gene, showed a remarkable sixfold increase in CHC cirrhosis. SARP3 belongs to the family of secreted frizzled-related proteins (sFRP) which are receptors in the Wnt pathway. Signal transduction in the Wnt pathway occurs through *β*-catenin which is responsible for the modulation of several basic cellular growth and differentiation signals [36]. Thus, SARP3 is thought to interfere with both apoptosis and hepatocellular growth [37]. More importantly, analysis of upregulated Wnt-associated genes in other forms of cirrhosis [38] revealed a possible unique role for SARP3 in CHC cirrhosis. Novel genes of yet poorly defined functionality in liver disease, such as ephrins, ephrin receptors, and Erb B2, were also overexpressed and were thought to reflect an increased hepatocellular turnover and remodeling which predisposes to malignant transformation, although further investigation is required. Overall, cDNA array analysis revealed a gene expression pattern consistent with chronic inflammation and greater hepatocellular apoptosis and proliferation; both studies on differential gene expression reveal a significant upregulation of proinflammatory, proapoptotic, and proproliferative genes in CHC cirrhosis, which is summarized in Table 1.

In a further attempt to investigate the complete pathway process for CHC, gene expression profile analysis at the cellular level, that is, hepatocytes and periportal lymphoid cells, also yielded an extensive list of upregulated genes for CHC. Interestingly, these genes were strongly related to immune response, lipid metabolism, and epidermal growth factor receptor (EGFR) signaling rather than apoptotic and fibrotic processes noted in CHB [39]. Similarly, the progression from CVH to HCC development significantly differs between CHB and CHC. In CHC, an impaired T cell response and induction of chemokines, cytokines, and growth factors that lead to carcinogenesis seem to play a more important role than the direct effect of HCV on the hepatocyte.

### 3.3. Regenerative Capacity of Hepatocytes in HCV-Mediated Cirrhosis

**Figure 1:**
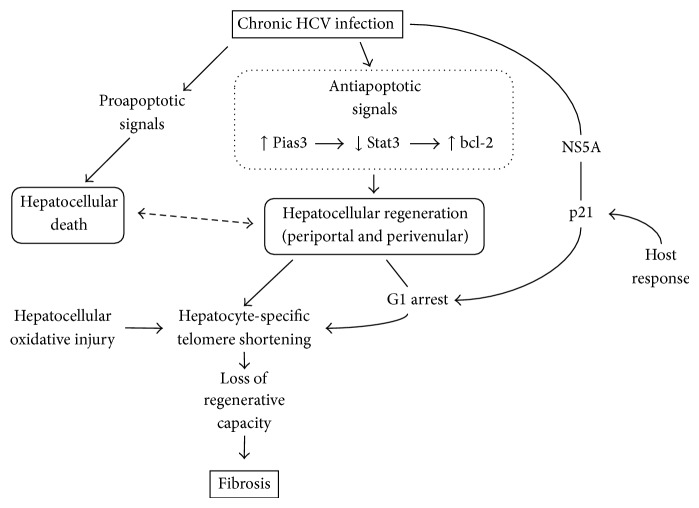
Outline of hepatocellular turnover in chronic HCV infection (see text for description). Stat: signal transducer and activator of transcription; Pias: protein inhibitor of activated Stat; NS5A: nonstructural 5A protein.

The regenerative capacity of liver tissue in response to chronic hepatocellular damage is directly related to the hepatocyte proliferative activity, an index used primarily for identifying cirrhotic patients at risk for developing HCC [40, 41]. An increased proliferative activity, however, is not sufficient* per se* to lead to normal regeneration if coordination of the molecular and cellular events is absent. An imbalance in hepatocyte turnover has been documented in several studies and seems to be responsible for deviation from normal regeneration in CHC and related cirrhosis, as outlined in this section.  Figure 1 depicts the effects of CHC on hepatocellular death and regeneration that ultimately lead to liver fibrosis.

#### 3.3.1. Hepatocyte Proliferative Activity in CHC and Cirrhosis

A significantly higher number of proliferating hepatocytes, detected by the Ki67 monoclonal antibody MIB1, have been identified in liver samples from patients with CHC and cirrhosis, compared to CHB [42]. Additionally, MIB1 labeling index correlated significantly with parameters indicative of the extent of liver damage, suggesting enhanced regeneration with higher disease activity. While in normal liver tissue hepatocellular proliferation occurs mainly in the periportal area [43], a significantly increased proliferative activity was observed in the intermediate and perivenular areas of the hepatic lobule in severe HCV-mediated liver injury. Apparently, CHC represents a potent noxious stimulus that forces the utilization of the full extent of hepatocellular reserve for the regenerative process. An increased proliferative activity of hepatocytes in CHC and related cirrhosis has been confirmed by digital image analysis of the distribution of proliferating cells compared to normal liver tissue. An antigen synthesized in the early G1 and S phases of the cell cycle, the proliferating cell nuclear antigen (PCNA) PC10, was detected immunohistochemically. PCNA expression was significantly increased in CHC and related cirrhosis, indicating a high proliferative activity, and was further increased in HCC liver samples [44]. Speculation that increased hepatocyte turnover in CHC may lead to HCC development, thus suggesting an indirect effect of HCV towards carcinogenesis, has also been made based upon these findings [45, 46]. The role of high hepatocellular proliferative activity in HCV-mediated cirrhosis has been further established in a statistical model correlating increased proliferation in advanced stages of HCV fibrosis and cirrhosis with the risk of HCC development. Cirrhotic patients with a high argyrophilic nucleolar organizer regions proliferation index (AgNOR-PI) showed an increased incidence of HCC and high AgNOR-PI was also associated with clinical and biochemical parameters pertaining to the severity of cirrhosis [47].

Despite the high proliferative activity exhibited by hepatocytes as a response to CHC-induced liver injury, regeneration does not follow the pattern observed in partial hepatectomy models in normal liver tissue. Specific molecular events seem to be responsible for the deviation from normal regenerative processes. Reentry of mature hepatocytes into the cell cycle as well as progression to proliferation is thought to be an essential step of liver regeneration, regardless of the cause of liver damage. In CHC, increased hepatocyte cell cycle entry has been confirmed using a novel marker, minichromosome maintenance protein 2 (Mcm-2) [48], a member of the prereplicative complex involved in permitting DNA replication. A unique feature of this family of proteins is their sensitivity to active cell cycle, since they rapidly degrade as the cell exits cycle [49]. Normal progression through the cell cycle is regulated by the sequential interaction of phase-specific cyclins and their respective cyclin-dependent kinases (cdk) [50]. The cycle phase distribution of hepatocytes during liver regeneration may differ depending on the etiologic factor of liver injury. CHC-induced liver injury has been associated with an increased hepatocyte turnover as shown by proliferation markers such as Ki67 [42], proliferating cell nuclear antigen [44], and Mcm-2 [48]. However, an arrest of proliferating hepatocytes at the G1 phase of the cell cycle with a small proportion of cells entering the S phase has been recently documented [51]. This finding was related to increased p21 expression in hepatocytes, which was in turn attributed to a direct viral effect through HCV protein NS5A [52] or a host response to HCV infection. Although the underlying mechanisms are not yet known, an impairment of liver regeneration effected by chronic HCV liver damage is obvious.

#### 3.3.2. Apoptosis and Hepatocyte Turnover in CHC and Cirrhosis

Evidence supporting predominance of antiapoptotic over proapoptotic pathways in CHC-induced liver injury has recently emerged. Although differential gene expression in HCV cirrhosis has revealed a proapoptotic gene profile [32, 33], a positive antiapoptotic balance through impaired Stat3 DNA-binding and Pias3 (protein inhibitor of activated Stat) upregulation has been documented in end-stage cirrhosis [53]. Signal transducer and activator of transcription 3 (Stat3) seems to play a role in regulating the apoptosis-proliferation balance by promoting cellular proliferation and by modulating the expression of proapoptotic proteins in animal experimental models [54] and human HCC [55]. Pias3 protein binds to activated Stat molecules resulting in inhibition of DNA-binding and subsequently inhibition of Stat-mediated gene activation [56, 57]. Although Stat3 expression and phosphorylation was not altered in HCV-mediated cirrhosis, a significantly weak Stat3 DNA-binding activity was observed in nuclear extracts from human cirrhotic liver samples with an associated upregulation of Pias3 expression. Inhibition of Stat3 signaling pathway resulted in overexpression of the antiapoptotic Bcl-2 gene with an associated shift towards hepatocellular proliferation [53]. These contradictory findings need to be further investigated; nevertheless, the significant role of apoptosis-regeneration balance in liver regeneration in end-stage CHC-induced liver injury is highlighted.

Increased cell-cycle turnover observed in HCV cirrhosis is not devoid of adverse effects on the hepatocyte. During the long-term alternation of hepatocellular death and regeneration, hepatocyte-specific telomere shortening progresses with each cell division [58]. This results in cellular senescence, that is, loss of regenerative capacity, and progression of liver fibrosis [59]. Sekoguchi et al. [60] showed that telomere shortening is more progressive in liver tissue with active cell-cycle turnover in patients with CHC and this is evident specifically in hepatocytes. Telomere shortening was also closely related to liver fibrosis and telomere shortening rate was significantly associated with the duration of CHC-mediated liver injury and the rate of fibrosis progression. The latter may be attributed to the loss of replicative capacity by hepatocytes and, at the same time, the maintenance of long telomeres in hepatic stellate cells, which do not participate in the regenerative process [61]. Furthermore, a possible role for hepatocellular oxidative injury, expressed by high-grade hepatic steatosis and high serum ferritin levels, in telomere shortening was investigated in the same group of patients. Current evidence supports that oxidative stress not only shortens telomeres but also may induce cellular senescence prematurely before telomeres are critically shortened [62]. Interestingly, in this study an acceleration of telomere shortening with subsequent hepatocellular senescence was observed in advanced stages of CHC.

## 4. Implications of New Antiviral Agents in CHC

Over the last decade, antiviral drug research has targeted the HCV life cycle and several “direct-acting antivirals” have been introduced in clinical practice. Inhibition of the HCV NS3-4A protease by telaprevir/boceprevir blocks polyprotein processing, thus inhibiting viral replication and possibly restoring interferon responsiveness. Nucleoside/nucleotide analogue inhibitors (NIs) of HCV RNA-dependent RNA polymerase, for example, sofosbuvir, and non-Nis (NNIs) are potent allosteric inhibitors of the HCV replicase complex, providing elimination of the viral load [63, 64]. The effect of these new treatments on chronic HCV-induced hepatocellular injury is yet unclear; however, the understanding of the underlying mechanisms is expected to change over the next few years.

## 5. Conclusion

CHC eventually leads to liver fibrosis and cirrhosis through a multistep, complex process involving hepatocyte death and regeneration. The pattern of enhanced cell-cycle turnover observed in HCV-mediated cirrhosis is regulated by multiple, not clearly understood, signaling pathways, an extensive network of cross talk molecules and a differential gene expression profile. The specific molecular and cellular events occurring in HCV cirrhosis have only recently been investigated, in an effort to guide research towards effective therapies of chronic infection that leads to HCC development. In the present review, the basic concepts and events pertaining to hepatocellular turnover in CHC-induced liver injury and cirrhosis were presented with focus on critical aspects of current research.

## Figures and Tables

**Table 1:** Apoptotic and antiapoptotic gene expression in chronic HCV-mediated liver injury and cirrhosis.

Gene/product	Function in CHC	Role
Apoptotic		
IFN-*α*	Upregulation	Cytokine
IRF-7	Upregulation	Transcription factor
Cytoplasmic dynein light chain	Upregulation	Motor protein, centrosome assembly
AML1/RUNX1	Upregulation	Transcription factor
Dr-nm23/NME3	Upregulation	Inhibition of granulocyte differentiation
Plasminogen activator inhibitor-2	Upregulation	Fibrinolysis, tissue repair
SARP3	Upregulation	Cellular growth and differentiation
CTGF	Upregulation	Tissue fibrosis, cell adhesion
CDKN1C	Upregulation	Tumor suppressor
CDC42	Downregulation	Cell cycle progression
Antiapoptotic		
Bcl-2	Upregulation	Cell proliferation, oncogenesis
Bcl-w	Upregulation	Cell proliferation
E2F transcription factor	Upregulation	Cell cycle progression
NF-*κ*B	Upregulation	Cell proliferation, regeneration
Tissue metalloproteinase inhibitor	Upregulation	Cell-cell interaction, anti-inflammatory cell response

CHC: chronic HCV-induced hepatitis; IFN: interferon; IRF: interferon regulatory factor; AML1: acute myeloid leukemia 1 protein; RUNX1: Runt-related transcription factor 1; NME3: nonmetastatic cells protein 3; SARP3: secreted apoptosis-related protein 3; CTGF: connective tissue growth factor; CDKN1C: cyclin-dependent kinase inhibitor 1C; CDC: cell division cycle 42 GTP-binding protein.
